# The Practice of Medicine by Statute

**Published:** 1868-02-15

**Authors:** 


### *The Practice of Medicine by Statute.*

The time-honored fable of the frogs who wanted a king, has been of much practical service to those who have duly pondered its meaning. The moral is put in more elegant phrase by Shakspeare, and, on the whole, it *is* well to consider whether it may not be better to submit to present ills rather than to fly to those we know not of. The trouble in the medical profession now (as it always has been) is, that it contains a class of uneasy gentlemen, with somewhat straitened intellects, who, hopeless of achieving any thing more than dull mediocrity by positive additions to the common stock of professional knowledge, seek to acquire notoriety at a cheaper rate by getting up a clamor about Medical Reform and Medical Legislation—"Notoriety is the next step to Fame"—and easily mistaken for it by the groundlings.

The JOURNAL reproduces with unqualified approval the following extract from an editorial in the *Chicago Post.* The editor, having spent many years in the profession, and many years of keen observation outside of it, knows whereof he writes, and is amply qualified to judge:

"If the diploma of a reputable school of medicine is not a sufficient passport to public confidence as an evidence of character and ability, it is not likely that a certificate from a State Board of Censors would be looked upon in a very different light. The element of politics would enter into their deliberations and influence their actions. People are notoriously suspicious of any thing that is done under the auspices of the opposite party, and a State doctor, whose qualifications were vouched for by a Republican Board of Censors would be obliged to seek his patients among his own partisans, and *vice versa.* The practical effect of such legislation as is contemplated, would be to place the whole business of the Board in the hands of that particular school of medicine which happened to wield the strongest political and financial influence.

The hardest thing for some politicians to understand is that there are some things which are outside the pale of legislation, and are best left to regulate themselves. This business of a State Medical Bureau is plainly a superfluity. Since no law can prevent people from patronizing quacks if they so desire, no amount of legislative wisdom can devise a scheme whereby the services of the right doctor shall in a majority of cases be secured."

We shall recur to this subject soon.

